# Supplementary material for: HIVprotI: an integrated web based platform for prediction and design of HIV proteins inhibitors
Source: J Cheminform. 2018 Mar 9;10:12. doi: 10.1186/s13321-018-0266-y (PMC5845081; doi:10.1186/s13321-018-0266-y)
Supplement: Supplementary file 2 — Additional file 2. Source code of HIVProtI web server. [file 13321_2018_266_MOESM2_ESM.zip › HIVprotI_Source-code/hivprotI/weka-3-6-6/doc/weka/experiment/LearningRateResultProducer.html]

LearningRateResultProducer


---


|  |  |  |
| --- | --- | --- |
| **Overview** \| **Package** \| **Class** \| **Tree** \| **Deprecated** \| **Index** \| **Help** \| **Weka's home** | |  |
| **PREV CLASS**   **NEXT CLASS** | **FRAMES**    **NO FRAMES**     **All Classes** |  |
| SUMMARY: NESTED \| FIELD \| CONSTR \| METHOD | DETAIL: FIELD \| CONSTR \| METHOD |  |


---


## weka.experiment Class LearningRateResultProducer

```
java.lang.Object
  weka.experiment.LearningRateResultProducer
```

**All Implemented Interfaces:**: java.io.Serializable, AdditionalMeasureProducer, OptionHandler, RevisionHandler, ResultListener, ResultProducer

---

``` public class LearningRateResultProducer extends java.lang.Object implements ResultListener, ResultProducer, OptionHandler, AdditionalMeasureProducer, RevisionHandler ```

Tells a sub-ResultProducer to reproduce the current run for varying sized subsamples of the dataset. Normally used with an AveragingResultProducer and CrossValidationResultProducer combo to generate learning curve results. For non-numeric result fields, the first value is used.


Valid options are:

```
 -X <num steps>
  The number of steps in the learning rate curve.
  (default 10)
```

```
 -W <class name>
  The full class name of a ResultProducer.
  eg: weka.experiment.CrossValidationResultProducer
```

```
 Options specific to result producer weka.experiment.AveragingResultProducer:
```

```
 -F <field name>
  The name of the field to average over.
  (default "Fold")
```

```
 -X <num results>
  The number of results expected per average.
  (default 10)
```

```
 -S
  Calculate standard deviations.
  (default only averages)
```

```
 -W <class name>
  The full class name of a ResultProducer.
  eg: weka.experiment.CrossValidationResultProducer
```

```
 Options specific to result producer weka.experiment.CrossValidationResultProducer:
```

```
 -X <number of folds>
  The number of folds to use for the cross-validation.
  (default 10)
```

```
 -D
 Save raw split evaluator output.
```

```
 -O <file/directory name/path>
  The filename where raw output will be stored.
  If a directory name is specified then then individual
  outputs will be gzipped, otherwise all output will be
  zipped to the named file. Use in conjuction with -D. (default splitEvalutorOut.zip)
```

```
 -W <class name>
  The full class name of a SplitEvaluator.
  eg: weka.experiment.ClassifierSplitEvaluator
```

```
 Options specific to split evaluator weka.experiment.ClassifierSplitEvaluator:
```

```
 -W <class name>
  The full class name of the classifier.
  eg: weka.classifiers.bayes.NaiveBayes
```

```
 -C <index>
  The index of the class for which IR statistics
  are to be output. (default 1)
```

```
 -I <index>
  The index of an attribute to output in the
  results. This attribute should identify an
  instance in order to know which instances are
  in the test set of a cross validation. if 0
  no output (default 0).
```

```
 -P
  Add target and prediction columns to the result
  for each fold.
```

```
 Options specific to classifier weka.classifiers.rules.ZeroR:
```

```
 -D
  If set, classifier is run in debug mode and
  may output additional info to the console
```

All options after -- will be passed to the result producer.

**Version:**
:   $Revision: 6425 $

**Author:**
:   Len Trigg (trigg@cs.waikato.ac.nz)

**See Also:**: Serialized Form

---

| **Field Summary** | |
| --- | --- |
| `static java.lang.String` | `STEP_FIELD_NAME`             The name of the key field containing the learning rate step number |


**Constructor Summary**

`LearningRateResultProducer()`


| **Method Summary** | |
| --- | --- |
| `void` | `acceptResult(ResultProducer rp, java.lang.Object[] key, java.lang.Object[] result)`             Accepts results from a ResultProducer. |
| `java.lang.String[]` | `determineColumnConstraints(ResultProducer rp)`             Determines if there are any constraints (imposed by the destination) on the result columns to be produced by resultProducers. |
| `void` | `doRun(int run)`             Gets the results for a specified run number. |
| `void` | `doRunKeys(int run)`             Gets the keys for a specified run number. |
| `java.util.Enumeration` | `enumerateMeasures()`             Returns an enumeration of any additional measure names that might be in the result producer |
| `java.lang.String` | `getCompatibilityState()`             Gets a description of the internal settings of the result producer, sufficient for distinguishing a ResultProducer instance from another with different settings (ignoring those settings set through this interface). |
| `java.lang.String[]` | `getKeyNames()`             Gets the names of each of the columns produced for a single run. |
| `java.lang.Object[]` | `getKeyTypes()`             Gets the data types of each of the columns produced for a single run. |
| `int` | `getLowerSize()`             Get the value of LowerSize. |
| `double` | `getMeasure(java.lang.String additionalMeasureName)`             Returns the value of the named measure |
| `java.lang.String[]` | `getOptions()`             Gets the current settings of the result producer. |
| `java.lang.String[]` | `getResultNames()`             Gets the names of each of the columns produced for a single run. |
| `ResultProducer` | `getResultProducer()`             Get the ResultProducer. |
| `java.lang.Object[]` | `getResultTypes()`             Gets the data types of each of the columns produced for a single run. |
| `java.lang.String` | `getRevision()`             Returns the revision string. |
| `int` | `getStepSize()`             Get the value of StepSize. |
| `int` | `getUpperSize()`             Get the value of UpperSize. |
| `java.lang.String` | `globalInfo()`             Returns a string describing this result producer |
| `boolean` | `isResultRequired(ResultProducer rp, java.lang.Object[] key)`             Determines whether the results for a specified key must be generated. |
| `java.util.Enumeration` | `listOptions()`             Returns an enumeration describing the available options.. |
| `java.lang.String` | `lowerSizeTipText()`             Returns the tip text for this property |
| `void` | `postProcess()`             When this method is called, it indicates that no more requests to generate results for the current experiment will be sent. |
| `void` | `postProcess(ResultProducer rp)`             When this method is called, it indicates that no more results will be sent that need to be grouped together in any way. |
| `void` | `preProcess()`             Prepare to generate results. |
| `void` | `preProcess(ResultProducer rp)`             Prepare for the results to be received. |
| `java.lang.String` | `resultProducerTipText()`             Returns the tip text for this property |
| `void` | `setAdditionalMeasures(java.lang.String[] additionalMeasures)`             Set a list of method names for additional measures to look for in SplitEvaluators. |
| `void` | `setInstances(Instances instances)`             Sets the dataset that results will be obtained for. |
| `void` | `setLowerSize(int newLowerSize)`             Set the value of LowerSize. |
| `void` | `setOptions(java.lang.String[] options)`             Parses a given list of options. |
| `void` | `setResultListener(ResultListener listener)`             Sets the object to send results of each run to. |
| `void` | `setResultProducer(ResultProducer newResultProducer)`             Set the ResultProducer. |
| `void` | `setStepSize(int newStepSize)`             Set the value of StepSize. |
| `void` | `setUpperSize(int newUpperSize)`             Set the value of UpperSize. |
| `java.lang.String` | `stepSizeTipText()`             Returns the tip text for this property |
| `java.lang.String` | `toString()`             Gets a text descrption of the result producer. |
| `java.lang.String` | `upperSizeTipText()`             Returns the tip text for this property |

**Methods inherited from class java.lang.Object**

`equals, getClass, hashCode, notify, notifyAll, wait, wait, wait`

**Field Detail**

### STEP\_FIELD\_NAME

```
public static java.lang.String STEP_FIELD_NAME
```

:   The name of the key field containing the learning rate step number


**Constructor Detail**

### LearningRateResultProducer

```
public LearningRateResultProducer()
```


**Method Detail**

### globalInfo

```
public java.lang.String globalInfo()
```

:   Returns a string describing this result producer

    :   **Returns:**: a description of the result producer suitable for displaying in the explorer/experimenter gui

---


### determineColumnConstraints

```
public java.lang.String[] determineColumnConstraints(ResultProducer rp)
                                              throws java.lang.Exception
```

:   Determines if there are any constraints (imposed by the
    destination) on the result columns to be produced by
    resultProducers. Null should be returned if there are NO
    constraints, otherwise a list of column names should be
    returned as an array of Strings.

    :   **Specified by:**: `determineColumnConstraints` in interface `ResultListener`
    :   **Parameters:**: `rp` - the ResultProducer to which the constraints will apply **Returns:**: an array of column names to which resutltProducer's results will be restricted. **Throws:**: `java.lang.Exception` - if constraints can't be determined

---


### doRunKeys

```
public void doRunKeys(int run)
               throws java.lang.Exception
```

:   Gets the keys for a specified run number. Different run
    numbers correspond to different randomizations of the data. Keys
    produced should be sent to the current ResultListener

    :   **Specified by:**: `doRunKeys` in interface `ResultProducer`
    :   **Parameters:**: `run` - the run number to get keys for. **Throws:**: `java.lang.Exception` - if a problem occurs while getting the keys

---


### doRun

```
public void doRun(int run)
           throws java.lang.Exception
```

:   Gets the results for a specified run number. Different run
    numbers correspond to different randomizations of the data. Results
    produced should be sent to the current ResultListener

    :   **Specified by:**: `doRun` in interface `ResultProducer`
    :   **Parameters:**: `run` - the run number to get results for. **Throws:**: `java.lang.Exception` - if a problem occurs while getting the results

---


### preProcess

```
public void preProcess(ResultProducer rp)
                throws java.lang.Exception
```

:   Prepare for the results to be received.

    :   **Specified by:**: `preProcess` in interface `ResultListener`
    :   **Parameters:**: `rp` - the ResultProducer that will generate the results **Throws:**: `java.lang.Exception` - if an error occurs during preprocessing.

---


### preProcess

```
public void preProcess()
                throws java.lang.Exception
```

:   Prepare to generate results. The ResultProducer should call
    preProcess(this) on the ResultListener it is to send results to.

    :   **Specified by:**: `preProcess` in interface `ResultProducer`
    :   **Throws:**: `java.lang.Exception` - if an error occurs during preprocessing.

---


### postProcess

```
public void postProcess(ResultProducer rp)
                 throws java.lang.Exception
```

:   When this method is called, it indicates that no more results
    will be sent that need to be grouped together in any way.

    :   **Specified by:**: `postProcess` in interface `ResultListener`
    :   **Parameters:**: `rp` - the ResultProducer that generated the results **Throws:**: `java.lang.Exception` - if an error occurs

---


### postProcess

```
public void postProcess()
                 throws java.lang.Exception
```

:   When this method is called, it indicates that no more requests to
    generate results for the current experiment will be sent. The
    ResultProducer should call preProcess(this) on the
    ResultListener it is to send results to.

    :   **Specified by:**: `postProcess` in interface `ResultProducer`
    :   **Throws:**: `java.lang.Exception` - if an error occurs

---


### acceptResult

```
public void acceptResult(ResultProducer rp,
                         java.lang.Object[] key,
                         java.lang.Object[] result)
                  throws java.lang.Exception
```

:   Accepts results from a ResultProducer.

    :   **Specified by:**: `acceptResult` in interface `ResultListener`
    :   **Parameters:**: `rp` - the ResultProducer that generated the results: `key` - an array of Objects (Strings or Doubles) that uniquely identify a result for a given ResultProducer with given compatibilityState: `result` - the results stored in an array. The objects stored in the array may be Strings, Doubles, or null (for the missing value). **Throws:**: `java.lang.Exception` - if the result could not be accepted.

---


### isResultRequired

```
public boolean isResultRequired(ResultProducer rp,
                                java.lang.Object[] key)
                         throws java.lang.Exception
```

:   Determines whether the results for a specified key must be
    generated.

    :   **Specified by:**: `isResultRequired` in interface `ResultListener`
    :   **Parameters:**: `rp` - the ResultProducer wanting to generate the results: `key` - an array of Objects (Strings or Doubles) that uniquely identify a result for a given ResultProducer with given compatibilityState **Returns:**: true if the result should be generated **Throws:**: `java.lang.Exception` - if it could not be determined if the result is needed.

---


### getKeyNames

```
public java.lang.String[] getKeyNames()
                               throws java.lang.Exception
```

:   Gets the names of each of the columns produced for a single run.

    :   **Specified by:**: `getKeyNames` in interface `ResultProducer`
    :   **Returns:**: an array containing the name of each column **Throws:**: `java.lang.Exception` - if key names cannot be generated

---


### getKeyTypes

```
public java.lang.Object[] getKeyTypes()
                               throws java.lang.Exception
```

:   Gets the data types of each of the columns produced for a single run.
    This method should really be static.

    :   **Specified by:**: `getKeyTypes` in interface `ResultProducer`
    :   **Returns:**: an array containing objects of the type of each column. The objects should be Strings, or Doubles. **Throws:**: `java.lang.Exception` - if the key types could not be determined (perhaps because of a problem from a nested sub-resultproducer)

---


### getResultNames

```
public java.lang.String[] getResultNames()
                                  throws java.lang.Exception
```

:   Gets the names of each of the columns produced for a single run.
    A new result field is added for the number of results used to
    produce each average.
    If only averages are being produced the names are not altered, if
    standard deviations are produced then "Dev\_" and "Avg\_" are prepended
    to each result deviation and average field respectively.

    :   **Specified by:**: `getResultNames` in interface `ResultProducer`
    :   **Returns:**: an array containing the name of each column **Throws:**: `java.lang.Exception` - if the result names could not be determined (perhaps because of a problem from a nested sub-resultproducer)

---


### getResultTypes

```
public java.lang.Object[] getResultTypes()
                                  throws java.lang.Exception
```

:   Gets the data types of each of the columns produced for a single run.

    :   **Specified by:**: `getResultTypes` in interface `ResultProducer`
    :   **Returns:**: an array containing objects of the type of each column. The objects should be Strings, or Doubles. **Throws:**: `java.lang.Exception` - if the result types could not be determined (perhaps because of a problem from a nested sub-resultproducer)

---


### getCompatibilityState

```
public java.lang.String getCompatibilityState()
```

:   Gets a description of the internal settings of the result
    producer, sufficient for distinguishing a ResultProducer
    instance from another with different settings (ignoring
    those settings set through this interface). For example,
    a cross-validation ResultProducer may have a setting for the
    number of folds. For a given state, the results produced should
    be compatible. Typically if a ResultProducer is an OptionHandler,
    this string will represent the command line arguments required
    to set the ResultProducer to that state.

    :   **Specified by:**: `getCompatibilityState` in interface `ResultProducer`
    :   **Returns:**: the description of the ResultProducer state, or null if no state is defined

---


### listOptions

```
public java.util.Enumeration listOptions()
```

:   Returns an enumeration describing the available options..

    :   **Specified by:**: `listOptions` in interface `OptionHandler`
    :   **Returns:**: an enumeration of all the available options.

---


### setOptions

```
public void setOptions(java.lang.String[] options)
                throws java.lang.Exception
```

:   Parses a given list of options.


    Valid options are:

    ```
     -X <num steps>
      The number of steps in the learning rate curve.
      (default 10)
    ```

    ```
     -W <class name>
      The full class name of a ResultProducer.
      eg: weka.experiment.CrossValidationResultProducer
    ```

    ```
     Options specific to result producer weka.experiment.AveragingResultProducer:
    ```

    ```
     -F <field name>
      The name of the field to average over.
      (default "Fold")
    ```

    ```
     -X <num results>
      The number of results expected per average.
      (default 10)
    ```

    ```
     -S
      Calculate standard deviations.
      (default only averages)
    ```

    ```
     -W <class name>
      The full class name of a ResultProducer.
      eg: weka.experiment.CrossValidationResultProducer
    ```

    ```
     Options specific to result producer weka.experiment.CrossValidationResultProducer:
    ```

    ```
     -X <number of folds>
      The number of folds to use for the cross-validation.
      (default 10)
    ```

    ```
     -D
     Save raw split evaluator output.
    ```

    ```
     -O <file/directory name/path>
      The filename where raw output will be stored.
      If a directory name is specified then then individual
      outputs will be gzipped, otherwise all output will be
      zipped to the named file. Use in conjuction with -D. (default splitEvalutorOut.zip)
    ```

    ```
     -W <class name>
      The full class name of a SplitEvaluator.
      eg: weka.experiment.ClassifierSplitEvaluator
    ```

    ```
     Options specific to split evaluator weka.experiment.ClassifierSplitEvaluator:
    ```

    ```
     -W <class name>
      The full class name of the classifier.
      eg: weka.classifiers.bayes.NaiveBayes
    ```

    ```
     -C <index>
      The index of the class for which IR statistics
      are to be output. (default 1)
    ```

    ```
     -I <index>
      The index of an attribute to output in the
      results. This attribute should identify an
      instance in order to know which instances are
      in the test set of a cross validation. if 0
      no output (default 0).
    ```

    ```
     -P
      Add target and prediction columns to the result
      for each fold.
    ```

    ```
     Options specific to classifier weka.classifiers.rules.ZeroR:
    ```

    ```
     -D
      If set, classifier is run in debug mode and
      may output additional info to the console
    ```

    All options after -- will be passed to the result producer.

    :   **Specified by:**: `setOptions` in interface `OptionHandler`
    :   **Parameters:**: `options` - the list of options as an array of strings **Throws:**: `java.lang.Exception` - if an option is not supported

---


### getOptions

```
public java.lang.String[] getOptions()
```

:   Gets the current settings of the result producer.

    :   **Specified by:**: `getOptions` in interface `OptionHandler`
    :   **Returns:**: an array of strings suitable for passing to setOptions

---


### setAdditionalMeasures

```
public void setAdditionalMeasures(java.lang.String[] additionalMeasures)
```

:   Set a list of method names for additional measures to look for
    in SplitEvaluators. This could contain many measures (of which only a
    subset may be produceable by the current resultProducer) if an experiment
    is the type that iterates over a set of properties.

    :   **Specified by:**: `setAdditionalMeasures` in interface `ResultProducer`
    :   **Parameters:**: `additionalMeasures` - an array of measure names, null if none

---


### enumerateMeasures

```
public java.util.Enumeration enumerateMeasures()
```

:   Returns an enumeration of any additional measure names that might be
    in the result producer

    :   **Specified by:**: `enumerateMeasures` in interface `AdditionalMeasureProducer`
    :   **Returns:**: an enumeration of the measure names

---


### getMeasure

```
public double getMeasure(java.lang.String additionalMeasureName)
```

:   Returns the value of the named measure

    :   **Specified by:**: `getMeasure` in interface `AdditionalMeasureProducer`
    :   **Parameters:**: `additionalMeasureName` - the name of the measure to query for its value **Returns:**: the value of the named measure **Throws:**: `java.lang.IllegalArgumentException` - if the named measure is not supported

---


### setInstances

```
public void setInstances(Instances instances)
```

:   Sets the dataset that results will be obtained for.

    :   **Specified by:**: `setInstances` in interface `ResultProducer`
    :   **Parameters:**: `instances` - a value of type 'Instances'.

---


### lowerSizeTipText

```
public java.lang.String lowerSizeTipText()
```

:   Returns the tip text for this property

    :   **Returns:**: tip text for this property suitable for displaying in the explorer/experimenter gui

---


### getLowerSize

```
public int getLowerSize()
```

:   Get the value of LowerSize.

    :   **Returns:**: Value of LowerSize.

---


### setLowerSize

```
public void setLowerSize(int newLowerSize)
```

:   Set the value of LowerSize.

    :   **Parameters:**: `newLowerSize` - Value to assign to LowerSize.

---


### upperSizeTipText

```
public java.lang.String upperSizeTipText()
```

:   Returns the tip text for this property

    :   **Returns:**: tip text for this property suitable for displaying in the explorer/experimenter gui

---


### getUpperSize

```
public int getUpperSize()
```

:   Get the value of UpperSize.

    :   **Returns:**: Value of UpperSize.

---


### setUpperSize

```
public void setUpperSize(int newUpperSize)
```

:   Set the value of UpperSize.

    :   **Parameters:**: `newUpperSize` - Value to assign to UpperSize.

---


### stepSizeTipText

```
public java.lang.String stepSizeTipText()
```

:   Returns the tip text for this property

    :   **Returns:**: tip text for this property suitable for displaying in the explorer/experimenter gui

---


### getStepSize

```
public int getStepSize()
```

:   Get the value of StepSize.

    :   **Returns:**: Value of StepSize.

---


### setStepSize

```
public void setStepSize(int newStepSize)
```

:   Set the value of StepSize.

    :   **Parameters:**: `newStepSize` - Value to assign to StepSize.

---


### setResultListener

```
public void setResultListener(ResultListener listener)
```

:   Sets the object to send results of each run to.

    :   **Specified by:**: `setResultListener` in interface `ResultProducer`
    :   **Parameters:**: `listener` - a value of type 'ResultListener'

---


### resultProducerTipText

```
public java.lang.String resultProducerTipText()
```

:   Returns the tip text for this property

    :   **Returns:**: tip text for this property suitable for displaying in the explorer/experimenter gui

---


### getResultProducer

```
public ResultProducer getResultProducer()
```

:   Get the ResultProducer.

    :   **Returns:**: the ResultProducer.

---


### setResultProducer

```
public void setResultProducer(ResultProducer newResultProducer)
```

:   Set the ResultProducer.

    :   **Parameters:**: `newResultProducer` - new ResultProducer to use.

---


### toString

```
public java.lang.String toString()
```

:   Gets a text descrption of the result producer.

    :   **Overrides:**: `toString` in class `java.lang.Object`
    :   **Returns:**: a text description of the result producer.

---


### getRevision

```
public java.lang.String getRevision()
```

:   Returns the revision string.

    :   **Specified by:**: `getRevision` in interface `RevisionHandler`
    :   **Returns:**: the revision


---


|  |  |  |
| --- | --- | --- |
| **Overview** \| **Package** \| **Class** \| **Tree** \| **Deprecated** \| **Index** \| **Help** \| **Weka's home** | |  |
| **PREV CLASS**   **NEXT CLASS** | **FRAMES**    **NO FRAMES**     **All Classes** |  |
| SUMMARY: NESTED \| FIELD \| CONSTR \| METHOD | DETAIL: FIELD \| CONSTR \| METHOD |  |


---
